# Supplementary material for: Effects of Open and Forest Habitats on Distribution and Diversity of Bumblebees (Bombus) in the Małopolska Upland (Southern Poland): Case Study
Source: Biology (Basel). 2021 Dec 3;10(12):1266. doi: 10.3390/biology10121266 (PMC8698831; doi:10.3390/biology10121266)
Supplement: Supplementary file 1 [file biology-10-01266-s001.zip › biology-1441114-supplementary.pdf]

Table S1: Twenty-five bumble bee (*Bombus*) species collected from mid-May to mid-September in natural and semi-natural habitats located in four parks (ŚPN, COLP, ChKLP, NLP) from Małopolska Upland (Southern Poland).

Species	Habitats	Parks	Months
B. barbutellus	natural	COLP, NLP, ChKLP	V-IX
B. bohemicus	natural, semi-natural	COLP, NLP, ChKLP	V-IX
B. campestris	natural, semi-natural	COLP, NLP, ChKLP	V-IX
B. confusus	natural	NLP, ChKLP, ŚPN	V-IX
B. hortorum	natural, semi-natural	COLP, NLP, ChKLP	IV-IX
B. humilis	natural	COLP. NLP, ChKLP	V-VIII
B. hypnorum	natural	COLP, NLP, ChKLP	V-VIII
B. jonellus	natural	COLP, NLP, ChKLP	V-VIII
B. lapidarius	natural, semi-natural	COLP, NLP, ChKLP	V-IX
B. lucorum	natural, semi-natural	COLP, NLP, ChKLP	V-IX
B. muscorum	natural	COLP, NLP, ChKLP	V-IX
B. norvegicus	natural, semi-natural	COLP, NLP, ChKLP	V-VIII
B. pascuorum	natural, semi-natural	COLP, NLP, ChKLP	V-IX
B. pomorum	natural	NLP. ŚPN	V-IX
B. pratorum	natural, semi-natural	COLP, NLP, ChKLP	V-VIII
B. ruderarius	natural, semi-natural	COLP, NLP, ChKLP	V-VIII
B. ruderatus	natural, semi-natural	ChKLP, ŚPN	V-IX
B. rupestris	natural, semi-natural	COLP, NLP, ChKLP	V-IX
B. semenoviellus	natural	COLP, NLP, ŚPN	V-VIII

B. soroeensis	natural	NLP, ŚPN	V-VIII
B. subterraneus	natural	NLP, ChKLP, ŚPN	IV-VIII
B. sylvarum	natural, semi-natural	COLP, NLP, ChKLP	IV-IX
B. sylvestris	natural, semi-natural	COLP, NLP, ChKLP	V-VIII
B. terrestris	natural, semi-natural	COLP, NLP, ChKLP	III-X
B. vestalis	natural, semi-natural	COLP, ChKLP, ŚPN	V-IX
